# Supplementary material for: DNA-Modified Liquid Crystal Droplets
Source: Biosensors (Basel). 2022 Apr 27;12(5):275. doi: 10.3390/bios12050275 (PMC9138460; doi:10.3390/bios12050275)
Supplement: Supplementary file 1 [file biosensors-12-00275-s001.zip › biosensors-1671087-supplementary.pdf]

biosensors

MDPI

Article

DNA-Modified Liquid Crystal Droplets

Xiuxiu Yang [1], Xiao Liang [1], Rajib Nandi [1], Yi Tian [1], Yiyang Zhang [1], Yan Li [1], Jingsheng Zhou [1], Yuanchen Dong [1], Dongsheng Liu [1], Zhengwei Zhong [2,*] and Zhongqiang Yang [1,*]

[1] Key Laboratory of Organic Optoelectronics and Molecular Engineering of the Ministry of Education, Department of Chemistry, Tsinghua University, Beijing 100084, China; yxx17@mails.tsinghua.edu.cn (X.Y.); liangxiao@tsinghua.edu.cn (X.L.); rajibnandi1987@gmail.com (R.N.); crystal_yi_work@163.com(Y.T.); yiyang.zhang@jiahua-china.com (Y.Z.); li_yan850603@163.com(Y.L.); zhoujingsheng@petrochina.com.cn (J.Z.); dongyc@iccas.ac.cn (Y.D.); liudongsheng@mail.tsinghua.edu.cn (D.L.)

[2] Department of Chemical Engineering, Hebei Petroleum University of Technology, Chengde 067000, China

* Correspondence: zhongzhengwei@cdpc.edu.cn (Z.Z.); zyang@tsinghua.edu.cn (Z.Y.)

Table S1. Details of the DNA sequences utilized in this paper.

Name	Detailed sequence information
R1	5'-TCTATTCGCATGAGAATTCCATTCACCG-TAAGTACACATCTACTTCACCA-3'
R2	5'-CTTACGGTGAATGGAATTCTCATGCGAATAGATA-CACATCTACTTCACCA-3'
DNA-C18	$C_{18}H_{37}(PO_3H)$-5'- TGGTGAAGTAGATGTGTA -3'
S1	5'-CCTGTCTGCCTAATGTGCGTCGTAAG TACACATCTACTTCACCA -3'
S2	5'-CTTACGACGCACAAGGAGATCATGAGCTGTCATCGGTCA -3'
S3	5'-CTCATGATCTCCTTTAGGCAGACAGGGACACACTAAGGT-3'
FAM-DNA	FAM-5′-TGACCGATGACAG-3'
ROX-DNA	ROX-5′-ACCTTAGTGTGTC-3'
Hg^{2+} aptamer 1	5'-CGCATTCAGGATTCTCAACTCGTATACACATCTACTTCACCA-3'
Hg^{2+} aptamer 2	5'-TTCGTGTTGTGTTTCCTGTTTGCGTACACATCTACTTCACCA-3'
Thrombin aptamer 1	5'-AGTCCGTGGTAGGGCAGGTT-GGGGTGACTTACACATCTACTTCACCA-3'
Thrombin aptamer 2	5'-GGTTGGTGTGGTTGGTACACATCTACTTCACCA-3'
ATP aptamer1	5'-TGGACACCTTCCTTACACATCTACTTCACCA-3' ACCTGTGGAAGGA
ATP aptamer2	5'-ACCTGGGGGAGTATTGCGAGGAAGAAGGTGTCACATA-CACATCTACTTCACCA-3′
H1	5'-TCTATTCGCATGAGGATCCCATTCACCG-TAAGTACACATCTACTTCACCA-3'
H2	5'-CTTACGGTGAATGGGATCCTCATGCGAATAGATA-CACATCTACTTCACCA-3'
random 1	5'-TCTATTCGCATGAGAATTGCATTCACCGTAAG-3'
random 2	5'-CTTACGGTGAATGCAATTGTCATGCGAATAGA-3'
C-DNA-SH	SH-5'-TACACATCTACTTCACCA-3'
NC-DNA-SH	SH-5'-GCTCACTCAGTCTCAACA-3'

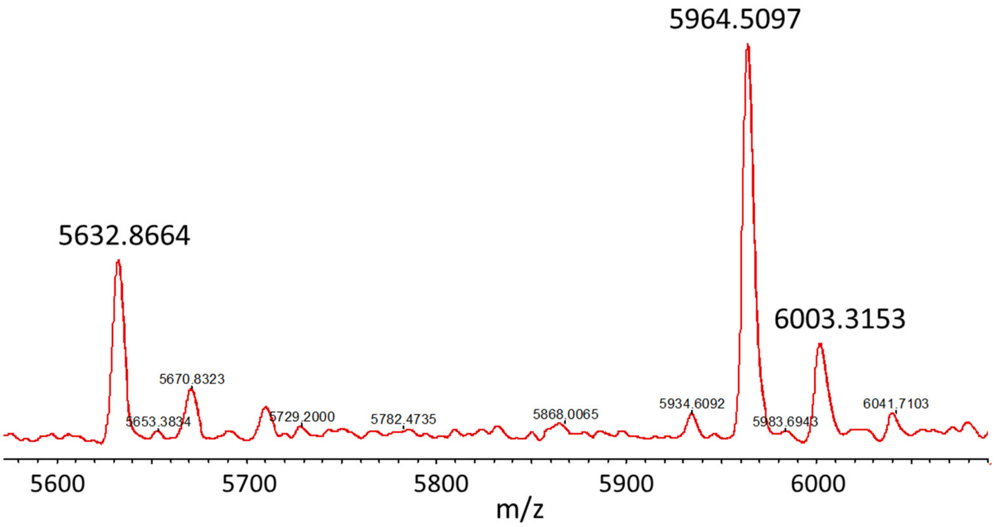

Figure S1. The MALDI - TOF mass spectra of DNA-C18.

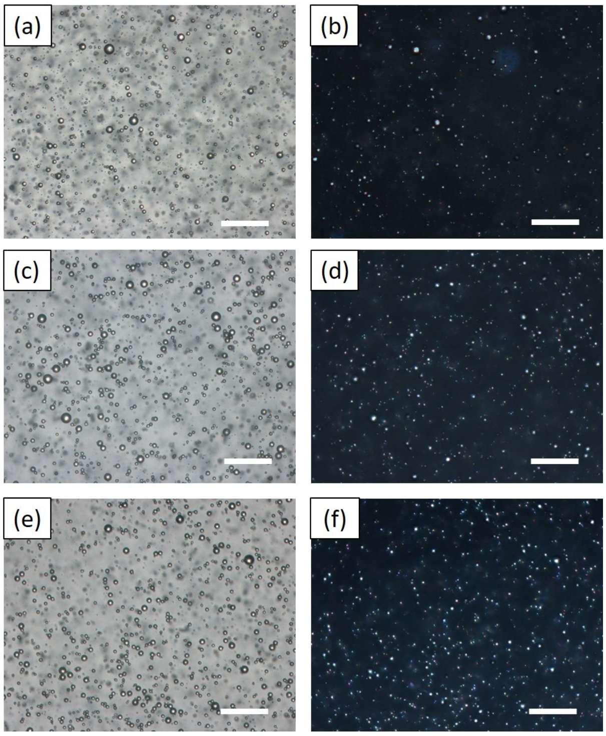

Figure S2. Microscopy images of DNA modified LC droplets before (a, b) and after adding (c, d) 20 μM R1, (e, f) 20 μM R2, (a, c, e) bright field, (b, d, f) polarized light images.

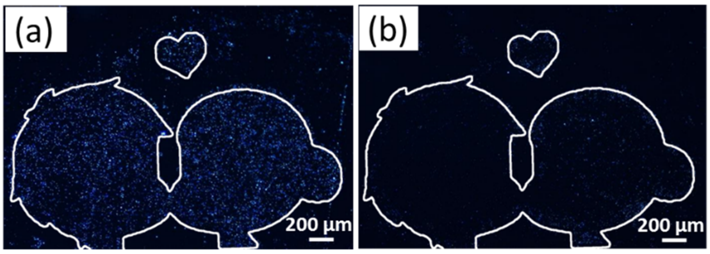

Figure S3. The printing pattern forming with LC droplets, which were linked to Au substrate by base complementation pairing rule. (a) LC droplets are linked to Au substrate by complementary DNA-SH, (b) LC droplets cannot be linked to Au substrate by non-complementary DNA-SH.

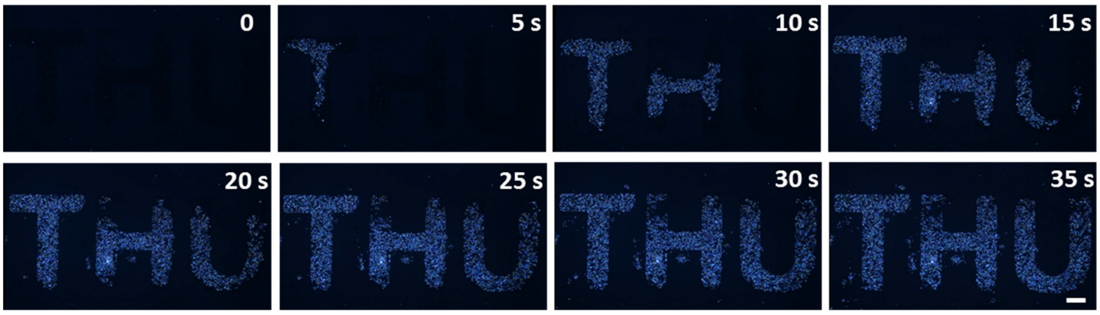

Figure S4. Patterned LC droplets were immersed in 40 °C PBS and the pattern change with the temperature decreased.

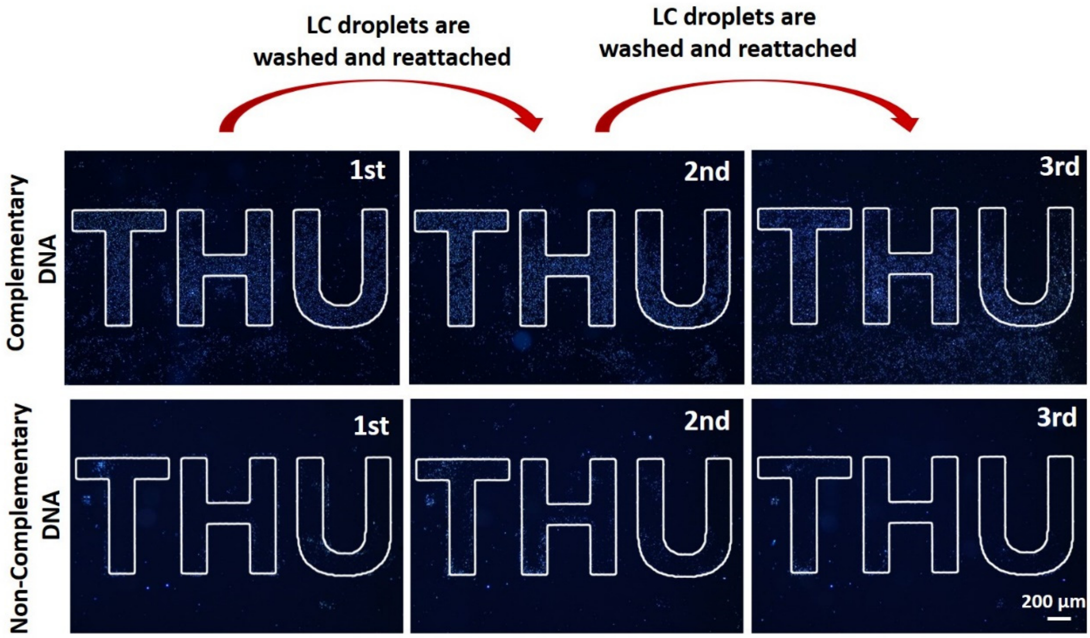

Figure S5. The Au substrate modified by C-SH-DNA and NC-DNA-SH after heating and washing which can be utilized to adsorb LC droplets at least 3 times.
